# Post-task Effects on EEG Brain Activity Differ for Various Differential Learning and Contextual Interference Protocols

**DOI:** 10.3389/fnhum.2018.00019

**Published:** 2018-01-31

**Authors:** Diana Henz, Alexander John, Christian Merz, Wolfgang I. Schöllhorn

**Affiliations:** Institute of Sport Science, University of Mainz, Mainz, Germany

**Keywords:** EEG, motor learning, differential learning, contextual interference, repetitive learning

## Abstract

A large body of research has shown superior learning rates in variable practice compared to repetitive practice. More specifically, this has been demonstrated in the contextual interference (CI) and in the differential learning (DL) approach that are both representatives of variable practice. Behavioral studies have indicate different learning processes in CI and DL. Aim of the present study was to examine immediate post-task effects on electroencephalographic (EEG) brain activation patterns after CI and DL protocols that reveal underlying neural processes at the early stage of motor consolidation. Additionally, we tested two DL protocols (gradual DL, chaotic DL) to examine the effect of different degrees of stochastic fluctuations within the DL approach with a low degree of fluctuations in gradual DL and a high degree of fluctuations in chaotic DL. Twenty-two subjects performed badminton serves according to three variable practice protocols (CI, gradual DL, chaotic DL), and a repetitive learning protocol in a within-subjects design. Spontaneous EEG activity was measured before, and immediately after each 20-min practice session from 19 electrodes. Results showed distinguishable neural processes after CI, DL, and repetitive learning. Increases in EEG theta and alpha power were obtained in somatosensory regions (electrodes P3, P7, Pz, P4, P8) in both DL conditions compared to CI, and repetitive learning. Increases in theta and alpha activity in motor areas (electrodes C3, Cz, C4) were found after chaotic DL compared to gradual DL, and CI. Anterior areas (electrodes F3, F7, Fz, F4, F8) showed increased activity in the beta and gamma bands after CI. Alpha activity was increased in occipital areas (electrodes O1, O2) after repetitive learning. Post-task EEG brain activation patterns suggest that DL stimulates the somatosensory and motor system, and engages more regions of the cortex than repetitive learning due to a tighter stimulation of the motor and somatosensory system during DL practice. CI seems to activate specifically executively controlled processing in anterior brain areas. We discuss the obtained patterns of post-training EEG traces as evidence for different underlying neural processes in CI, DL, and repetitive learning at the early stage of motor learning.

## Introduction

A large body of research has shown increased motor learning rates in different variable practice approaches compared to repetitive learning protocols (for an overview see Lage et al., 2015). However, variable training is interpreted in a versatile manner. While traditionally biggest learning success was expected by numerous repetitions a single to-be-learned movement (Gentile, 1972), the variability of practice theory (Moxley, 1979) suggested to stabilize an automatized movement only by repeating the invariant elements of a to-be-learned movement in combination with numerous variable parameters in accordance to Schmidt’s (1975) schema theory. Almost in parallel another approach provided evidence for improved learning results by not only focusing on the single to-be-learned task but rather by letting learn at least a second task in parallel (Shea and Morgan, 1979). Proponents of this approach could show that training of discrete tasks in an interleaved order often induces increased motor learning mainly in fine motor tasks after interfered acquisition compared with practicing these tasks in a repetitive order (Brady, 2008). This phenomenon is called the contextual interference (CI) effect and was first observed and examined in the field of cognitive learning, namely verbal learning (Battig, 1966) before having been transferred to the learning of fine motor tasks (Shea and Morgan, 1979). In CI learning the context of motor learning is manipulated by practicing several defined tasks in either a repetitive (blocked) order or an interleaved (random) order. Hereby the to-be-learned tasks have to be performed in a prescribed, correct manner, deviations here from are considered as errors and typically have to be avoided (Shea and Zimny, 1983).

A different theoretical perspective on variable practice was introduced on the background of the system dynamic approach (Haken, 1970; Glansdorff and Prigogine, 1971; Haken et al., 1985). While previous learning approaches refer to deviations from a predefined ideal state rather in a destructive way the system dynamic theory considers them more as constructive fluctuations, a more neutral term that is derived from stochastic physics. Hence systems that are in energetic exchange with the environment are characterized to show fluctuations continuously. In addition, a transition from one stable state to another in such systems is typically accompanied by an increase of fluctuations that causes a period of instability. During such a phase transition these systems can be interpreted as a kind of exploring a variety of modes in order to find new and even more effective states. These phenomena have been observed and described extensively in different areas of sports and everyday movements (Kelso, 1995; Davids et al., 2006). Instead of considering the increase of fluctuations as a passive ontological phenomenon of dissipative systems, the differential learning (DL) approach suggests to take advantage of the increased fluctuations as an active instrument in order to lead the system towards a zone of instability where less energy is needed for achieving a new state (Schöllhorn, 1999, 2000; Schöllhorn et al., 2006, 2010).

### The DL Approach

With increasing fluctuations by adding stochastic perturbations to a to-be-learned movement the DL approach initiates a self-organized learning process in which the learner will find an individually optimized solution for the movement problem. Thereby the actual to-be-learned movement must no more be repeated again in its ideal form (Schöllhorn, 2016). Because of the continuous differences between the subsequent movements in the DL approach no augmented feedback and no repetition is recommended. Whereas the CI approach tries to achieve performance improvement by adding a context to the to-be-learned movement in form of interrupting the acquisition sequence of the to-be-learned movement with a second or third concrete movement. In this case all three movements would be repeated several times and similarly often during the whole acquisition process. More concretely, in case of learning a tennis forehand stroke the DL approach suggests to execute the forehand first with extended elbow, then with flexed elbow, then with stiff knees, another time with a large of short distance to the ball or with left arm in front of the trunk or on the back, etc. In contrast the CI approach suggests to mix the forehand acquisition with a back hand stroke or with a serve stroke in a more or less random order.

Current research shows increased acquisition rates in DL compared to repetitive learning. This was demonstrated for football (Schöllhorn et al., 2004; Hegen and Schöllhorn, 2012), handball (Wagner and Müller, 2008), basketball (Schönherr and Schöllhorn, 2003; Lattwein et al., 2014), track and field (Jaitner et al., 2003; Beckmann and Schöllhorn, 2006; Beckmann and Gotzes, 2009), volleyball (Römer et al., 2009), and tennis (Humpert and Schöllhorn, 2006). DL shows increased acquisition rates as well as increased rates in motor learning (Beckmann and Schöllhorn, 2006; Savelsbergh et al., 2010). Evidence for the beneficial effects of DL on postural sway is shown in a study by James (2014). A detailed overview is given by Beckmann (2013). In a recent clinical study, stroke patients underwent an occupational training intervention in a DL and a repetitive learning protocol. The DL group showed a more effective course of arm recovery compared to the group that underwent repetitive training (Repšaitė et al., 2015). Meanwhile this approach has been adopted by sports pedagogy as nonlinear pedagogical approach (Chow et al., 2006).

These numerous verifications lead to an unification of the most common motor learning approaches under the umbrella of noise resulting in the model of stochastic resonance (Schöllhorn et al., 2006, 2009). Thereby each learning approach is considered to be accompanied by a certain amount and structure of fluctuations. Most intriguingly, Gebkenjans et al. (2007) were able to show that the random adding of a second movement to a first tennis serve during the acquisition phase in accordance to the CI approach leads to a greater increase of fluctuations of the first tennis serve than by a blocked protocol. Consequently, this brought up the question whether the found learning progress in CI is rather due to the provoked increase of fluctuations in the to-be-learned movement than caused by the context of other concrete movements. Up to an optimal amount of noise an increase of the learning rate is expected, beyond, a further increase of fluctuations is detrimental for the learning rate. On basis of an ongoing scientific discussion a distinction between gradual and chaotic DL was introduced (Schöllhorn, 2016). While gradual DL is characterized by systematic and mostly expectable changes between two subsequent movements the chaotic DL approach is accompanied by rather unexpected and unpredictable variations from one movement to the next. Exemplarily, a gradual DL strategy would expect changes in the left foot ankle joint followed by changes of the right ankle joint that are followed by changes of the right knee joint, etc. In contrast, the chaotic DL approach would follow with changes of the right elbow after have been started with the left foot ankle joint, and this would be followed by changes in the left shoulder joint and the left hand joint. Beside the search for an optimal design of exercises on the phenomenological level the mechanisms on the neurophysiological level are of main interest.

### Neurophysiological Evidence for Improved Motor Learning in CI and DL

Several studies have shown evidence for the differences in the underlying neurophysiological processes in interleaved over repetitive learning protocols that lead to superior performance on the long term. Neurophysiological studies on CI training show increased activity in the MI area after CI training. Increased neural activity during interleaved over repetitive practice might be a suitable explanation for the beneficial effects of CI. Tanaka et al. (2005) showed differences in electroencephalographic (EEG) brain activation in CI and a repetitive learning protocol. Serrien (2009) demonstrated enhanced interhemispheric connectivity in motor learning of bimanual finger tapping when sequences were practiced in an interleaved order. In the same manner, it was shown that CI practice enhances connectivity in the fronto-parietal networks (Lin et al., 2013). These results indicate that interleaved practice is beneficial for the formation of memory traces and efficient long-term retrieval. An fMRI study showed differences in excitability of the M1 area in interleaved and repetitive practice dependent on age (Lin et al., 2012). As a brain behavior correlate Lin et al. (2011) demonstrated a higher excitability of the M1 area in interleaved practice. In a previous fMRI study, Wymbs and Grafton (2009) found neural correlates for effects of offline learning. They showed activation in the premotor-parietal network. Further, sensorimotor and subcortical regions were activated during preparation and retention after a randomized training design. A different pattern of results occurred after blocked practice. Here, for both preparation and reproduction of the movements brain was activated in the ipsilateral left motor cortex. Cross et al. (2007) showed in a fMRI study increased activity in sensorimotor and premotor regions during variable practice compared to blocked practice. These areas are associated with motor preparation, sequencing, and response selection. The observed patterns of activation are in line with the argumentation that CI leads to superior performance in a sequencing task due to increased capacity to prepare and select motor responses.

In the present study, we compared immediate post-training effects of different variable practice protocols, and a repetitive protocol in badminton serve training on spontaneous EEG brain activity. To date, the relation between motor learning and post-task resting state EEG is not fully understood. Previous research has indicated that immediate post-task effects might represent a neural substrate for the early stage of motor consolidation. Studies in humans revealed that learning leaves local traces immediately after task performance (Tanaka et al., 2011; Buschkuehl et al., 2012; Crupi et al., 2013). Recent studies showed that post-task traces are task-specific and local. For instance, Hung et al. (2013) demonstrated task-specific increases in theta power in parieto-occipital areas after a driving simulation game. Similar results were obtained in movement adaptations to a rotated display (Ghilardi et al., 2000; Krakauer et al., 2000; Huber et al., 2004; Perfetti et al., 2011). Changes in alpha activity in resting-state EEG were found in areas that showed EEG changes during task performance (Landsness et al., 2011; Perfetti et al., 2011). Moisello et al. (2013) demonstrated changes in spontaneous EEG following 40-min training on a sequence-learning task. They concluded that sequence learning is related to changes in theta and alpha power in frontal and posterior areas during task performance. Increases in alpha activity were obtained in occipito-parietal areas after task performance. The obtained post-task changes reflect learning processes and are discussed as a correlate for neural plasticity (Moisello et al., 2013). Further, imaging studies using O15-PET showed activations in frontal and parieto-occipital areas after spatial sequence learning (Ghilardi et al., 2000, 2003; Nakamura et al., 2001). Summarizing, these studies indicate that there is a parallel between motor learning and post-task resting EEG.

A recent study examined immediate post-task effects after differential badminton serve training (Henz and Schöllhorn, 2016). Badminton players performed serves in a repetitive learning protocol and in a DL protocol with a high degree of variations. Results showed increases in EEG theta activity in frontal and central regions with increases in alpha activity in central and posterior regions after DL compared to repetitive learning and also relative to a baseline resting condition. In a consecutive study, effects of differencial soccer goal shooting compared to repetitive training on EEG brain activity were tested (Henz et al., 2014). The effects on EEG brain activity were replicated: results showed increased central and posterior theta and posterior and central alpha activity. Additionally, mental practice of the DL protocol did not cause the increase in EEG theta and alpha activity that was obtained after active DL. In a further study, different patterns of brain activations occurred after DL, CI, and repetitive training of the badminton serve. Results showed increased EEG frontal-midline theta activity as well as posterior and central alpha activity after DL, whereas post-training effects after CI displayed increased beta and gamma activity in anterior areas (Henz et al., 2015).

The main aim of the present study was to compare immediate post-task effects of the DL and CI protocol to fathom the underlying neural mechanisms at the early stage of motor learning. We investigated the effects of three variable training protocols, one according to the CI approach, the second one according to the DL approach with a smaller amount of noise which we named the gradual DL condition, and the third one with a bigger amount of noise which we named the chaotic DL condition. In the CI condition, trials were performed in an interleaved order with two varying movements. As shown in a previous pilot study on the post-training effects of CI and DL (Henz et al., 2015), we hypothesized that differences in post-task brain activation patterns would occur in repetitive learning, CI, and DL. DL is characterized to have high affordances on motor control and stimulates the motor and somatosensory system to a high degree. From this, we expected that EEG theta and alpha activity in somatosensory and motor areas would increase after both DL conditions in line with the findings of a previous study (Henz and Schöllhorn, 2016). We argue that theta and alpha activity in somatosensory and motor areas indicate motor learning processes (Moisello et al., 2013) and are enhanced in DL due to a tighter stimulation of the somatosensory and motor system. Further, we hypothesized increased somatosensory theta activity and increased alpha activity in the motor areas after chaotic DL compared to gradual DL. We expected that chaotic DL would activate the motor and somatosensory areas of the brain to a higher degree due to increased stochastic fluctuations during the motor learning sequence in form of a greater sum of differences in subsequent movements in comparison to smaller subsequent differences in gradual DL, CI, or repetitive learning. In contrast, we expected increases in beta and gamma activity that indicate executive cognitive controlled processing in anterior regions after CI according to the results of previous studies (Henz et al., 2015; Henz and Schöllhorn, 2016).

## Materials and Methods

### Participants

Twenty-two beginners in badminton (mean age 23.2 years, age range 18–32, 16 males, 6 females) participated in the study. Subjects were recruited from badminton courses at the University of Mainz, Germany. None of the subjects had current neurological diseases or a history of neurological impairments or intake of medication that may have influenced EEG brain activity. All subjects gave written informed consent. The study was approved by the local ethics committee of the University of Mainz. All experimental procedures complied with the standards of the Helsinki Declaration of the World Medical Association Assembly. All subjects were naïve as to the purpose of the study.

### EEG Recording Details

EEG brain activity was recorded from 19 electrodes that were placed according to the international 10–20 system on the scalp with reference to the nose. EEG signals were recorded from the electrodes Fp1, Fp2, F3, F7, Fz, F4, F8, C3, Cz, C4, T3, T4, P3, P7, Pz, P4, P8, O1, O2. Electrodes are referred to the cortices in this study as follows: frontal cortex (Fp1, Fp2, F3, F7, Fz, F4, F8), motor cortex (C3, Cz, C4), temporal cortex (T3, T4), parietal cortex (P3, P7, Pz, P4, P8), and occipital cortex (O1, O2). The Micromed Brainquick amplifier (SD–LTM–32) and Micromed Brainspy software (Micromed, Venice, Italy) were used for the EEG recordings. Impedances of all electrodes were kept at 10 kΩ or below. EEG data were recorded continuously and digitized at a sampling rate of 256 Hz. EEG signals were amplified with a time constant of 0.3 s (high pass filter: 0.5 Hz; low pass filter: 120 Hz; frequency range: 0.5–120 Hz). To assess electrooculographic (EOG) data two electrodes were placed at the medial upper and lateral orbital rim of the right eye. EOG signals were amplified with a time constant of 0.3 s (high pass filter: 0.1 Hz; low pass filter: 120 Hz; frequency range: 0.5–120 Hz). Heart rate was measured continuously as a control variable using a Polar watch (Polar A 300).

### Experimental Procedure

Prior to the experiment the experimental tasks were explained. Each subject was explained where and how to stand, the appropriate grip for each serve, and how to move. Initial performance in badminton serves measured in terms of a hit ratio was assessed on the day before the training intervention. For the initial test, 50 badminton serves were performed towards a target placed at the left service court. The target was placed at a distance of 8.40 m from the players. Badminton serves were performed from the service line of the right court. On the consecutive day, EEG was measured before and after the training interventions. Participants began with a resting condition. Spontaneous EEG was recorded for 4 min with eyes-open. Then, subjects performed one of the experimental conditions that had a duration of 20 min. Immediately after each experimental condition, a 4 min resting baseline EEG sequence was recorded with eyes-open. The study contained four experimental conditions. Badminton serves were performed according to a repetitive, CI, and two DL (gradual, chaotic) training protocols in a randomized within-subjects design. Subjects performed serves with the right hand into a target placed test on the left service court. The target was placed at a distance of 8.40 m from the players. The position of the target was the same as in the initial performance test. In the repetitive learning condition, all serves were performed with the forehand without movement variations. In the CI condition, subjects performed the serves alternating with the fore- and backhand in serial order. In the gradual DL condition, serves were performed in three blocks, which comprised trials with variations of either one, two, or three movement parameters. In gradual DL, trials were performed with blocks of variation of one, two, or three movement parameters. In case of one parameter the athletes were instructed to change only a single joint position or movement for the subsequent execution whereas in case of two or three parameters the corresponding number of joint positions or movements was asked to be changed for the next movement. Exemplarily, with one parameter e.g., they were instructed to do the badminton serve with left foot in front, subsequently they should do the movement with the left arm in front before they did the movement with an extended right elbow. In case of two parameters the subjects were asked to do two of these instructions at once and correspondingly the same for three parameters. In case of gradual DL it was systematic from changes in the foot joint over changes of the knee and hip joint towards the trunk, head and arm joints as well as from changes of 1–3 parameters. Whereas in case of chaotic DL the sequence was random for joints and number of parameters. The movements in the gradual DL condition were the same as in the chaotic DL condition just in different order. In both DL conditions, practice trials were repeated for a maximum of three times. The number of overall badminton serves was set to 90 badminton serves in each experimental condition with a training duration of 20 min. EEG data were recorded during the five resting conditions: (1) pre-training rest; (2) post-repetitive training rest; (3) post-CI rest; (4) post-gradual DL rest; and (5) post-chaotic DL rest that were used for subsequent statistical analyses. After each post-training recording interval subjects had a 5 min break.

### EEG Analysis

Spontaneous EEG was assessed with eyes-open. Four minute sequences were recorded before, and after each experimental learning condition. Spontaneous EEG was not segmented. EEG and EOG data were visually inspected and data portions containing artifacts resulting from eye movements and muscle movements were removed. Additionally, independent component analyses (ICAs) were performed for the EEG signal and components that resulted from artifacts were removed. For the analysis of the EEG data Fast Fourier Transforms were performed to calculate the mean power spectra for the theta (4–7.5 Hz), alpha (8–13 Hz), beta (14–30 Hz), and gamma (31–40 Hz) bands.

### Hit Ratios

Hit ratios of badminton serves were determined for the initial test, and for each experimental condition (repetitive training, CI, gradual DL, chaotic DL) as behavioral measure. Based on a basic set of 90 badminton serves per experimental condition, the score of hits that were placed into the target was measured.

### Statistical Analysis

Means and standard deviations of the hit ratios of badminton serves were calculated for the initial test. Additionally, Cronbach’s alpha was determined as a measure of internal consistency. Mauchly’s test of sphericity was calculated to test the assumptions of repeated-measures analysis of variance (ANOVAs) for the hit ratios and the EEG data. Consecutive ANOVAs were performed when the *p-*values were equal or exceeded 0.05. A one-way repeated-measure ANOVA that included the within-subjects factor training (repetitive training, CI, gradual DL, chaotic DL) was performed for the hit ratio of each of the subsequent training interventions. In a consecutive step, data was subjected to *post hoc t*-tests with Bonferroni correction. For the EEG data, repeated-measure ANOVAs were performed separately for the theta, alpha, beta, and gamma bands that included the within-subject factors as experimental condition (baseline rest, repetitive training, CI, gradual DL, chaotic DL), and location (Frontal, Central, Temporal, Parietal, Occipital). In a consecutive step, *post hoc* *t-tests* with Bonferroni correction were calculated for significant main or interaction effects. Statistical significance of the tests was achieved when the *p*-values were less than 0.05.

## Results

### Performance Errors

**Figure 1:**
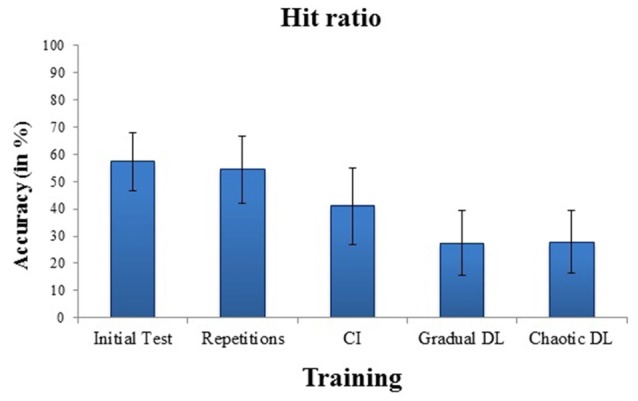
Mean and SDs of performance errors in the initial test, and after repetitive training, contextual interference (CI), gradual differential learning (DL), and chaotic DL.

Means and standard deviations of hit ratios for badminton serves for the initial test and for the training conditions are depicted in Figure 1. High internal consistency was obtained for the initial test, *α* = 0.87. The ANOVA showed a highly significant effect for training, *F*_(3,63)_ = 6.43, *p* = 0.001, $\eta_{\text{p}}^{2}$ = 0.23. *Post hoc* tests revealed increased hit ratios in repetitive training, compared to CI, *p* = 0.001, gradual DL, *p* < 0.001, and chaotic DL, *p* < 0.001. No significant difference was obtained between gradual DL, and chaotic DL.

### Spontaneous EEG

**Figure 2:**
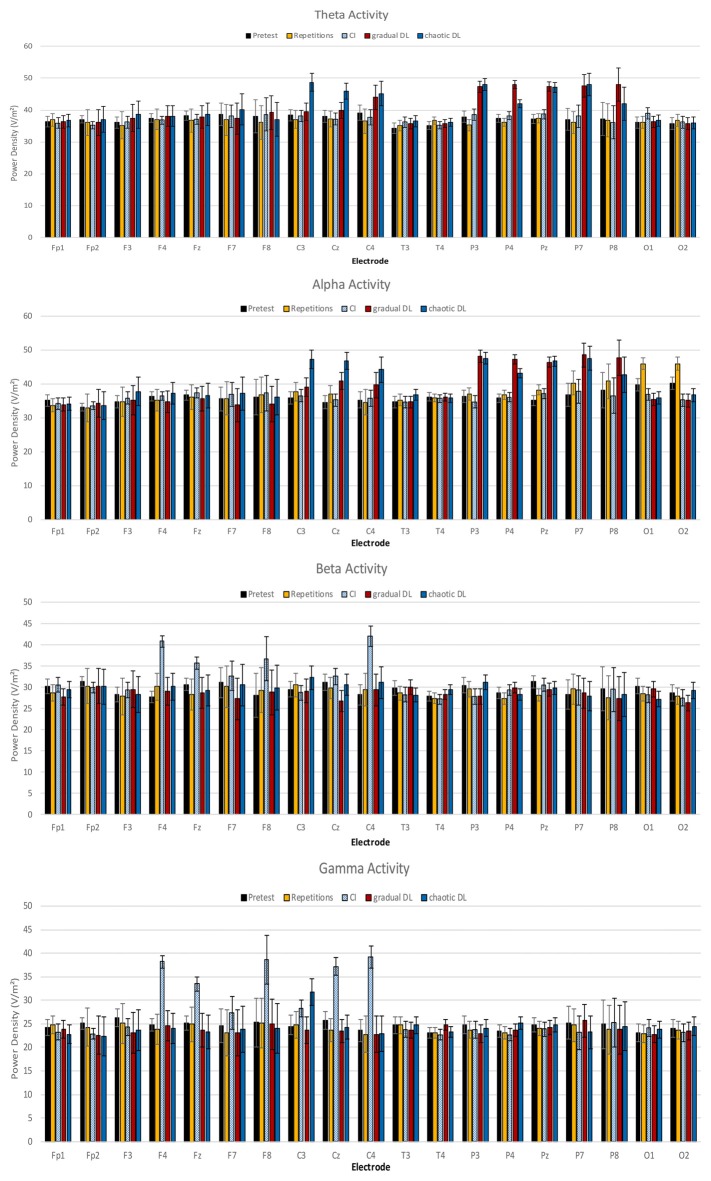
Means and SDs of power densities of spontaneous electroencephalographic (EEG) brain activity at baseline, and after CI, gradual DL, chaotic DL, and repetitive training.

Mean power spectra for the EEG theta, alpha, beta, and gamma bands are depicted in Figure 2. The ANOVA of theta activity showed a significant main effect for experimental condition, *F*_(4,84)_ = 3.16, *p* = 0.018, $\eta_{\text{p}}^{2}$ = 0.13. *Post hoc* tests revealed increased overall theta activity in gradual DL compared to CI, repetitive training, and baseline rest, *p* = 0.02. Further results showed increased theta activity in gradual DL compared to CI, *p* = 0.03, repetitive training, *p* = 0.02, and baseline rest. The ANOVA for the factor locations demonstrated a highly significant main effect, *F*_(4,84)_ = 4.14, *p* = 0.004, $\eta_{\text{p}}^{2}$ = 0.165. A significant condition × location interaction was obtained, *F*_(16,336)_ = 1.80, *p* = 0.030, $\eta_{\text{p}}^{2}$ = 0.08. *Post hoc* tests revealed increased theta activity at parietal electrodes in chaotic DL compared to CI, *p* = 0.02, repetitive training, *p* = 0.03, and baseline rest, *p* = 0.04. Further, theta activity was increased at central electrodes in gradual DL compared to CI, *p* = 0.02, repetitive training, *p* = 0.02, and baseline rest, *p* = 0.03. Finally, results showed increased theta activity at parietal electrodes in chaotic DL compared to gradual DL, *p* = 0.04, CI, *p* = 0.02, repetitive training, *p* = 0.03, and baseline rest, *p* = 0.02.

The ANOVA of alpha activity showed a significant main effect for the factor experimental condition, *F*_(4,84)_ = 3.20, *p* = 0.019, $\eta_{\text{p}}^{2}$ = 0.13. *Post hoc* tests revealed increased overall alpha activity in gradual DL compared to CI, *p* = 0.02, repetitive learning, *p* = 0.03, and baseline rest, *p* = 0.03. Further, alpha activity was increased in chaotic DL compared to CI, *p* = 0.02, repetitive learning, *p* = 0.02, and baseline rest, *p* = 0.03. The ANOVA for the factor location showed a significant main effect, *F*_(4,84)_ = 2.97, *p* = 0.024, $\eta_{\text{p}}^{2}$ = 0.12. A significant condition × location interaction was identified, *F*_(16,336)_ = 1.90, *p* = 0.020, $\eta_{\text{p}}^{2}$ = 0.13. *Post hoc* tests showed increased alpha activity at occipital electrodes in repetitive training compared to CI, *p* = 0.03, gradual DL, *p* = 0.02, chaotic DL, *p* = 0.03, and baseline rest, *p* = 0.03. In chaotic DL, alpha activity was significantly increased at central electrodes compared to repetitive training, *p* = 0.03, CI, *p* = 0.03, gradual DL, *p* = 0.04, and baseline rest, *p* = 0.03. Further, results showed increased alpha activity at parietal electrodes in chaotic DL compared to CI, *p* = 0.02, repetitive training, *p* = 0.03, and baseline rest, *p* = 0.03.

The ANOVA of beta activity revealed a significant main effect for experimental condition, *F*_(4,84)_ = 2.87, *p* = 0.028, $\eta_{\text{p}}^{2}$ = 0.12. *Post hoc* tests revealed that overall beta activity was increased in CI compared to gradual DL, *p* = 0.02, chaotic DL, *p* = 0.02, repetitive training, *p* = 0.04, and baseline rest, *p* = 0.03. The ANOVA for the factor location showed a significant main effect, *F*_(4,84)_ = 2.68, *p* = 0.037, $\eta_{\text{p}}^{2}$ = 0.11. *Post hoc* tests revealed increased beta activity in CI at central electrodes compared to frontal, *p* = 0.04, temporal, *p* = 0.02, and occipital electrodes, *p* = 0.03.

The ANOVA of gamma activity showed a significant main effect for experimental condition, *F*_(4,84)_ = 3.07, *p* = 0.021, $\eta_{\text{p}}^{2}$ = 0.14. *Post hoc* tests revealed increased overall gamma activity in CI compared to chaotic DL, *p* = 0.02, repetitive training, *p* = 0.03, gradual DL, *p* = 0.03, and baseline rest, *p* = 0.02. The ANOVA for the factor locations showed a highly significant main effect, *F*_(4,84)_ = 3.50, *p* = 0.01, $\eta_{\text{p}}^{2}$ = 0.15. A significant experimental condition × location interaction was obtained, *F*_(16,336)_ = 1.86, *p* = 0.023, $\eta_{\text{p}}^{2}$ = 0.082. *Post hoc* comparisons showed increased gamma activity in frontal electrodes in CI compared to gradual DL, *p* = 0.03, chaotic DL, *p* = 0.03, and repetitive training, *p* = 0.03. Further, gamma activity was increased at central electrodes in CI compared to gradual DL, *p* = 0.03, chaotic DL, *p* = 0.03, repetitive training, *p* = 0.03, and baseline rest, *p* = 0.02.

## Discussion

This is the first study that compares immediate post-task EEG changes following repetitive learning, CI, and DL. Results clearly demonstrate distinguishable patterns of post-training EEG brain activation in repetitive learning, CI, and DL. After gradual DL and chaotic DL overall theta and alpha activity increased, whereas increased central and parietal beta and gamma activity was demonstrated after CI. Comparing gradual DL and chaotic DL, no difference was found in overall theta and alpha activity between gradual DL and chaotic DL. In chaotic DL, central and parietal alpha activity was increased compared to gradual DL. Finally, increased occipital alpha activity was increased after repetitive learning.

Our results confirm findings from previous neurophysiological investigations on post-training effects of DL on brain activity (Henz et al., 2014; Henz and Schöllhorn, 2016). These studies demonstrated increases in fronto-central midline theta activity and central alpha activity. Further, findings from a previous EEG study on acute post-training effects of CI, DL and repetitive learning protocols were replicated (Henz et al., 2015).

The obtained patterns of post-task EEG brain activations indicate different neural processes immediately after the CI and DL protocols. In the following sections, we discuss different lines of interpretations of the patterns of post-task brain activities in the experimental conditions and reasons for improved motor learning in CI and DL.

### Different Brain Areas Are Activated after CI and DL

Neurophysiological studies on the effect of CI interventions indicate that the M1 area and the DLPFC are activated. Activation of the DLPFC indicates involvement of executively controlled cognitive processes (Cross et al., 2007; Wymbs and Grafton, 2009; Lin et al., 2011, 2012). The results of the present study are in line with this hypothesis: we observed increased activations in the gamma frequency band in the frontal regions after CI which indicates that executively controlled cognitive processes are engaged. In contrast, results indicate that motor and somatosensory information processing plays a key role in DL while activation in the frontal cortex is down regulated towards increased theta frequencies. Additionally, the degree of stochastic fluctuations as tested in the comparison of gradual DL and chaotic DL modulates activity in the somatosensory and motor areas with increased activations in these areas in chaotic DL. In a recent study, it was shown that reduced frontal cortical activity resulted from increases in somatosensation during walking (Clark et al., 2014). A further argument is derived from the assumptions of the transient hypofrontality hypothesis (Dietrich, 2006) that was subsequently tested in studies on the effect of an aerobic exercise on cognitively controlled processes (e.g., Davranche et al., 2015; Soga et al., 2015). One main reason is that controlled cognitive processing is reduced during continued gross motor activity. This effect is caused by an overt stimulation of the visual, somatosensory, and motor areas in exercise that afford allocation of metabolic resources to these brain areas at the cost of metabolic processes in the frontal cortex.

### Post-task Effects Are Frequency-Specific in CI and DL

Literature has shown that both variable practice protocols lead to increased motor learning rates compared to repetitive practice. The differences in post-task EEG brain activation patterns following CI and DL indicate different neural pathways of information processing that contribute to increases in motor learning. As gamma activation is found in selective attentional processing (Engel and Singer, 2001; Varela et al., 2001), we argue that the structure of the CI protocol enforces a style of motor learning that is characterized by tight executive cognitive controlled processing. In contrast, increased parietal and central theta and alpha activity in DL indicate that processing of somatosensory and motor information plays the key role at this early stage of motor learning. The DL protocol strongly enhances sensory integration from different modalities (motor, visual, haptic, proprioceptive, verbal instructions etc.) compared to CI and repetitive learning. Therefore, one line of argumentation is that increases in theta (Kanayama et al., 2015) activity are correlates for multisensory integration in DL. Considering increases in alpha activity after DL, EEG studies support the notion that coherence is increased between mono-sensory brain areas during processing of cross-modal tasks. For instance, Hummel and Gerloff (2005) showed increases in alpha activity in participants with good performance in a cross-modal matching task. The authors argue that the obtained EEG-coherence is a measure for the synchronization of brain areas that are tightly related to cross-modal integration. Further, Classen et al. (1998) demonstrated increased EEG coherence between the visual and the somatosensory regions as well as between the visual and the motor regions when a visuomotor tracking task was performed. Finally, in a recent study it was shown that the occurrence of alpha and mu frequency band in brain areas that are related to motor learning is a relevant predictor for motor performance (Meyer et al., 2014). From this point of view, we argue that DL reinforces the development of a multisensory movement representation that leads to increased motor learning rates. As a further consequence, this multisensory movement representation might lead to increased stability of the movement representation.

### Error Processing in DL Activates Working Memory Processes

Resource allocation is one of the cognitive processes located within the model of working memory. It has been shown that resource allocation plays an important part in processing of movement errors. This argumentation is in line with the reinvestment theory (Masters and Maxwell, 2008). One basic assumption is that errors in motor performance force individuals to reinvest cognitive resources for movement control. In the present study, lowest performance errors were reached in repetitive learning, followed by CI. Highest performance errors were obtained in gradual and chaotic DL. No significant difference was observed between both DL conditions. In a recent study by Lam et al. (2010) it was shown that the outcome of the previous trial had a substantial influence on the amount of resources allocated to programming the following movement when motor tasks were repeated multiple times. One substantial finding is that more resources were allocated if the previous movement trial contained an error. We argue that increased affordances on error processing necessitates increased working memory capacities. Activation of somatosensory and motor areas after DL in the theta range indicate working memory processes (Carretié, 2001; Mitchell et al., 2008; Myers et al., 2014; Tóth et al., 2014) and are a neurophysiological correlate for encoding of new information (Klimesch et al., 1996, 1997; Klimesch, 1999; Bastiaansen et al., 2002).

One limitation to this study that we address is the problem to compare task difficulties of DL and CI. One reason derives from the underlying theoretical assumptions of the CI and DL approach. Variability of practice theory (Moxley, 1979) suggested to stabilize an automatized movement only by repeating the invariant elements of a to-be-learned movement in combination with numerous variable parameters in accordance to Schmidt’s (1975) schema theory. In contrast, the CI approach provided evidence for improved learning results by not only focusing on the single to-be-learned task but rather by letting learn at least a second task in parallel (Shea and Morgan, 1979). In the present study, in DL combinations of variable movement parameters were performed. In contrast, CI comprised as the to-be-learned task the forehand movement that was contrasted by the backhand movement.

### Post-task EEG Traces Indicate Consolidation Processes at the Early Stage of Motor Learning

The obtained post-task effects on brain activity are interpreted as a neural substrate for the early stage of motor memory consolidation. Recent studies on motor learning have demonstrated changes in EEG alpha power after motor adaptation to a rotated display (Huber et al., 2004; Ghilardi et al., 2009; Perfetti et al., 2011). Moisello et al. (2013) showed post-task changes in spontaneous EEG brain activity after sequence learning. Post-learning EEG recordings showed increased alpha power in right occipito-parietal areas relative to the resting EEG at pretest. The authors conclude that learning processes are correlated with changes in frontal theta activity and increases of alpha activity in occipito-parietal areas. These post-task changes in EEG activity may reflect a trace of learning and neural substrate of use-dependent plasticity in sequence learning (Moisello et al., 2013). As there was no retention test in the present study, interpretations of the post-task EEG brain activation patterns are limited to the post-task interval of 4 min. In this context, the relation between motor learning and post-task resting state EEG could be made more explicit with a correlational analysis for learning rates within the practice intervals and post-task EEG activations in future work. Further, brain activity during and after task performance should be assessed to compare the neural processes. Finally, studies will elucidate the mid- and long-term effects of CI and DL practice on brain activity.

The results of our study have important implications for the design of training and therapeutic interventions, for instance in achievement sports and rehabilitative settings. Further studies will investigate the temporal course and the long-term effects of DL on the underlying neurophysiological processes of motor learning.

## Conclusion

The results of the present study reveal distinguishable immediate post-training effects on EEG brain activity for different training protocols within the variable practice approach. After gradual DL and chaotic DL theta and alpha activity increased, whereas after CI increased beta and gamma activity in anterior regions was obtained. Further, after chaotic DL, central and parietal alpha activity was increased compared to gradual DL. These findings suggest different neural processes at the early stage of motor learning in DL and CI with executively cognitive controlled processing in CI and somatosensory and motor consolidation in DL.

## Author Contributions

The authors DH, AJ, CM and WIS cooperated on preparing the manuscript.

## Conflict of Interest Statement

The authors declare that the research was conducted in the absence of any commercial or financial relationships that could be construed as a potential conflict of interest.
